# Religiosity Decline in Europe: Age, Generation, and the Mediating Role of Shifting Human Values

**DOI:** 10.1007/s10943-022-01670-x

**Published:** 2022-09-23

**Authors:** Maciej Koscielniak, Agnieszka Bojanowska, Agata Gasiorowska

**Affiliations:** Institute of Psychology, SWPS University of Social Sciences and Humanities, ul. Kutrzeby 10, 61-719 Poznan, Poland

**Keywords:** Religiosity, Human values, Generations, Age

## Abstract

Despite the fact that age is associated with higher religiosity, the aging European population has experienced a noticeable religiosity decline over recent decades. This study aimed to explain this paradox and to link it to an intergenerational shift in the pattern of values (as conceptualized by Shalom Schwartz). We conducted extended mediation analyses on the relationships between generational affiliation and the level of personal religiosity via human values in two studies (European Social Survey round 7, *N* = 29,775; and European Social Survey rounds 1–9, *N* = 224,314). Our results confirm a pronounced trend of religiosity decline and explain this process by changes in personal values. In particular, Europe's generational increase in openness to change values explains religiosity decline above and beyond the effect of people’s developmental age. We conclude that the perspective of human values provides a significant rationale for further research on religiosity, in relation to both past and future generations of Europeans.

**Supplementary Information:**

The online version contains supplementary material available at 10.1007/s10943-022-01670-x.

## Introduction

European societies are aging (Vancea & Solé-Casals, 2016), and age has been shown to be positively correlated with religiosity. If developmental mechanisms connected to aging robustly explain religiosity levels, then these aging societies should have become increasingly religious over the course of the past century. However, this was not the case. In fact, a significant decline in religiosity in European countries has been observed over the past decades (Argue et al., 1999; Deaton, 2009; Firebaugh & Harley, 1991; Hout & Greeley, 1987). Therefore, it seems that there must be another factor responsible for religiosity decline that works above and beyond mere age. In this study, we hypothesized that one factor that has significant potential to explain this decline is generational affiliation. Generations are more than just groups of people of similar age; generations can be defined as communities of shared experiences, feelings (Mannheim, 1940, 1970), and shared values (Marcus et al., 2017; Twenge et al., 2012). Because values and religiosity are significantly related (Chan et al., 2018; Hitlin & Piliavin, 2004; Kuşdil & Akoğlu, 2014; Maio, 2010; Rohan, 2000; Saroglou et al., 2004), generational differences in values should translate into generational differences in religiosity. Therefore, we propose that religiosity decline can be explained by values differing across generations, and that this mechanism is mostly independent of the age effect.

### Religiosity and Aging

Religiosity is usually defined as an organized system of beliefs, practices, rituals, and symbols related to a higher power (Paterson & Francis, 2017, p. 2; see also: Han & Richardson, 2010; Kidwai et al., 2014; Lavretsky, 2010). It serves different psychological needs. For example, it soothes existential anxiety in the face of the inevitability of death (Pyszczynski et al., 2010) and helps provide cognitive closure and reduces perceived uncertainty (Duriez, 2003; Saroglou, 2002). Religiosity is governed by the underlying processes of identity and motivation. Along with social identity theory (Tajfel & Turner, 1979), religiosity is a significant element of human identity, which regulates a person’s self-concept (“I am a religious person”) and their group belonging (with those who practice the same religion; Van Cappellen et al., 2016). It can be expressed and strengthened through individual and social religious practices. The practices that are carried out alone (e.g., praying) express a person’s self-concept, and they are more likely to be a sign of an intrinsic religious motivation (as opposed to extrinsic; Allport & Ross, 1967), whereas the practices that are carried out with others (e.g., attending religious services) strengthen social bonds and are also representative of social processes.

Although generational affiliation is the key focus of this study, the aspect of age in explaining religiosity levels cannot be ignored. Numerous studies have demonstrated a significant relationship between age and religiosity, with older people being systematically more religious than people from younger groups (Argue et al., 1999; Shulgin et al., 2019; Wilmoth et al., 2015). Most researchers explain the age and religiosity relationship by referring to developmental mechanisms connected to aging and claim that as individuals get older, they become more involved in religious practices and are more likely to identify as religious (Bahr, 1970; Barker et al., 1992; Davie & Vincent, 1998).

Theoretical explanations for the developmental mechanism behind the age-religiosity relationship can be derived from Terror Management Theory (for overviews, see: Kesebir & Pyszczynski, 2012; Pyszczynski et al., 2010) and Socioemotional Selectivity Theory (Carstensen et al., 1999; Löckenhoff & Carstensen, 2004). Terror Management Theory is based on the idea that humans, unlike other animals, are sophisticated enough in their mental abilities to be aware of the fragility of life and the inevitability of ultimate death, and proposes that the awareness of mortality has the potential to generate paralyzing anxiety and that the management of this potential anxiety is essential for effective functioning (Pyszczynski et al., 2010). According to the theory, people develop an anxiety buffering system that, as long as it is functional, protects against existential anxiety, and provides psychological equanimity. Religiosity may also have this function, and therefore may become increasingly important as death gets closer (Jonas & Fischer, 2006) because it serves as emotional compensation and increases well-being toward the end of life (McCoy et al., 2000). Socioemotional selectivity theory proposes that as their time horizons become shorter, people tend to change their priorities and focus more on positive events and cues than on negative ones (Löckenhoff & Carstensen, 2004). Religion may provide these positive stimuli, and its social aspects may help to increase a sense of community, leading to decreased anxiety, increased well-being, longevity, and somatic health (Abdel-Khalek et al., 2019; Cox & Hammonds, 1989; Hall, 2006; Saleem & Saleem, 2020).

Because people become more religious with age and, at the same time, societies are aging (Vancea & Solé-Casals, 2016), we should be able to observe an increase in religiosity over the past decades. However, what we have recently witnessed is actually a clear decline in religiosity (Shulgin et al., 2019; Voas & Chaves, 2016). A possible explanation for this decline is that the observed relationship between age and religiosity is a statistical artifact associated with cohort replacement (Argue et al., 1999): older people represent earlier generations and it is their generational affiliation that explains their religiosity levels and not mere age. If this is true, then we should be looking at the characteristics of each subsequent generation that could explain religiosity decline, and not only at developmental mechanisms connected to age. One such pattern underlying religiosity decline may be the intergenerational shift in human values.

### Religiosity and Basic Human Values

In the psychological sense, values are beliefs or cognitive representations of motivations and goals (Schwatz, 1992). They constitute the core of human identity and serve as cross-situational standards or criteria for evaluating behaviors and decisions (Rokeach, 1973). According to Schwartz’s model (Schwatz, 1992), 10 basic values—self-determination, stimulation, hedonism, achievement, power, security, conformity, tradition, benevolence, and universalism—exist in a circular structure that determines the relationships between them (positively related or conflicting). This perspective also allows for the grouping of basic values into higher-order dimensions: openness to change (self-determination, stimulation, and hedonism), self-enhancement (achievement and power), conservation (security and tradition), and self-transcendence (benevolence and universalism) (Schwartz et al., 2001; Sortheix & Schwartz, 2017). Openness to change and self-enhancement represent personal focus, expressing individual needs and attitudes, whereas self-transcendence and conservation represent social focus, regulating how a person relates to others. Furthermore, openness to change and self-transcendence represent self-expansive and growth values, whereas self-enhancement and conservation represent self-protective, anxiety-based values (Saroglou et al., 2004; Schwartz et al., 2001).

The relationship between values and religiosity is twofold. First, specific religious traditions relate to individual value hierarchies (Schwartz & Huismans, 1995) because these two areas are strongly intertwined as they fall within a broader framework of personal and social identity. Being religious means upholding specific values (“I am Christian = I hold Christian values”) and specific religious traditions dictate which values people are supposed to uphold and how they should function within society (e.g., Weber, 2013). Indeed, empirical evidence confirms that socialization with respect to religion can be viewed as a process of social transmission of values (Saroglou et al., 2004).

Second, and more pertinent to this paper, the level of religiosity can be reinforced by some values and weakened by others, regardless of a specific religious tradition, because psychologically some values play a similar role to religiosity. Specifically, values falling into the dimension of conservation, such as tradition and security, are rooted in anxiety reduction and uncertainty avoidance (Schwatz, 1992; Sortheix & Schwartz, 2017), so they serve psychological functions similar to religiosity (Duriez, 2003; Greenberg et al., 1986; Saroglou, 2002). Other values, such as conformity and benevolence, have social functions similar to those of religious identity, consistent with social identity theory (Tajfel & Turner, 1979).

Moreover, religiosity is primarily identified with faith in delayed reward (eternal life in exchange for temporal duties). Therefore, the values associated with gratification "here and now" (e.g., hedonism) have a negative association with religious beliefs (Schwartz & Huismans, 1995). Values that are most commonly expressed through behaviors inconsistent with the requirements dictated by most religious traditions—engaging in sensory or bodily pleasures (hedonism), seeking out novelty (stimulation) or freedom and independence in choosing one’s life goals (self-direction) (Bardi & Schwartz, 2003)—are difficult to align with religious doctrines. It is therefore natural that higher levels of openness to change are related to lower religiosity*.* Indeed, empirical evidence confirms that religiosity is positively associated with tradition and conformity, and to a lesser extent, with security and benevolence. It is also negatively associated with hedonism, stimulation, and self-direction, and, to a lesser extent, with achievement, power, and universalism (Chan et al., 2018; Hitlin & Piliavin, 2004; Kuşdil & Akoğlu, 2014; Maio, 2010; Rohan, 2000; Saroglou et al., 2004). These findings seem robust across different contexts (religious denominations, countries, etc.). Therefore, in this study, we also expect religiosity to be related mainly to high endorsement of conservation values and low endorsement of openness to change values. We also predict that the relationship between religiosity and self-transcendence and self-enhancement values will be weaker or even absent.

### Generational Affiliation, Shifts in Values, and Religiosity Decline

The term “generation” signifies more than just being born at a specific time within a specific culture; it also denotes shared life experiences, attitudes, goals, and ways of interpreting reality (Mannheim, 1970). For example, the term “Generation X” (Coupland, 1991) is used to describe people born in the 1960s or 1970s for whom career and material wealth are of key importance. Their aspirations in the spiritual domain are low. Tapscott (2009) suggested that people’s generational affiliations can be determined by the media that was dominant when they were at the peak of their cognitive and social functioning. After the Silent Generation, or Matures, (born between 1928 and 1945), there are three post-World War II generations: Baby Boomers (born between 1946 and 1964), for whom radio and television were the key media; Generation X (born between 1965 and 1976), who experienced a shift in the dominant media; Generation Y, or Millennials, (born between 1977 and 1997), whose mindset was formed mostly by the Internet, and Generation Z (born after 1998).

Distinctions between generations based on year of birth are quite arbitrary, but there is no doubt that to some extent, they define groups of people who share common values and are aware of their uniqueness with respect to other groups. For example, the Matures, who experienced the Great Depression and World War II, are considered to be hardworking and devoted, and willing to sacrifice for their loved ones, conform to tradition, and accept authority (Strauss & Howe, 1992). Their values are therefore conservation rather than openness to change and self-transcendence rather than self-enhancement (Lyons et al., 2007). Baby Boomers, who were born and raised in the era of economic prosperity in the post-war period, had to compete for attention in their childhood and for jobs as they entered the job market, but they also grew up with a sense of entitlement, expecting prosperity and satisfaction in their lives. They are said to be self-indulgent, hedonist, pleasure-seeking, and achievement oriented; however, they share a concern for society and are perceived as optimistic idealists (Lyons et al., 2007; Strauss & Howe, 1992). Compared with Matures, they are more likely to endorse openness to change values, be less attached to conservation values, and be similar to the previous generation in terms of self-transcendence and self-enhancement (Lyons et al., 2007).

Generation Xers were raised in the age of economic uncertainty. They experienced increased parental divorce rates and grew up in single-parent families much more often than members of previous generations (Howe & Strauss, 2009). They are often described as highly cynical, severely independent, and entrepreneurial (Lyons et al., 2007; Wey Smola & Sutton, 2002), preferring to rely on their own efforts rather than expecting help from others, favoring change over stability and tradition, and self-enhancement over self-transcendence (Howe & Strauss, 2009; Lyons et al., 2007). Like Generation Xers, Millennials are characterized as independent, innovative, adaptable, and change-oriented; however, they are considered more optimistic and self-absorbed than Generation Xers (Tapscott, 2009).

Using the Schwartz Value Survey, Lyons et al. (2007) demonstrated that the two younger generations, Generation Xers and Millennials, value self-transcendence and conservation less than the two older generations, Matures and Baby Boomers. The same pattern had been observed in earlier research that examined age rather than generation; age was positively correlated with conservation (tradition, conformity, and security) and self-transcendence (benevolence and universalism) and negatively correlated with openness to change (self-direction, stimulation, and hedonism values) and self-enhancement (power and achievement values) (Schwartz et al., 2001). Hence, it seems difficult to unravel the effects of age and generation in cross-sectional research. However, some longitudinal studies and meta-analyses have suggested that people and societies are shifting toward more individualistic values and away from social values. Over the past few years, various studies have demonstrated increasing levels of individualism (Marcus et al., 2017; Twenge, 2014), self-enhancement, extrinsic values (Kasser, 2016; Twenge & Campbell, 2014; Twenge et al., 2012) and agentic orientation (Twenge, 1997), but decreasing levels of intrinsic values (Twenge et al., 2012), empathy (Konrath et al., 2011) and secure attachment styles (Konrath et al., 2014). Most of these studies draw their data from Western or highly educated urban samples; hence, value changes observed in these studies may be explained by the fact that as societies become more prosperous, they also become less traditional (Bakan, 1966). This global shift toward individualistic, extrinsic, and self-enhancement values (Kasser, 2016; Marcus et al., 2017) has also been connected to the development of new media, such as the Internet, underlining the fact that more recent generations are born and raised in different societal contexts than the earlier ones, and are not just younger (Tapscott, 2009).

**Fig. 1:**
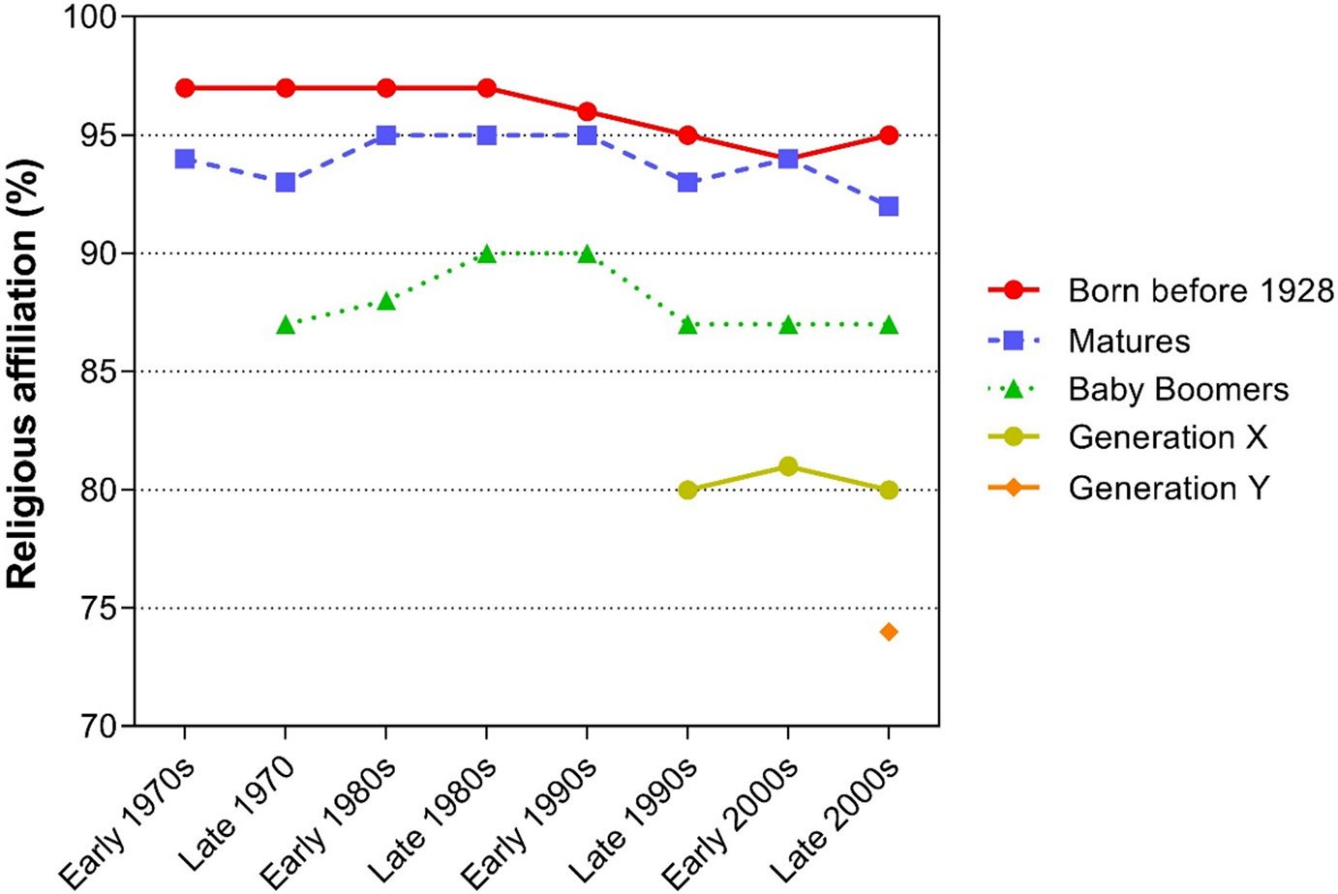
Changes over time in religious affiliation in successive European generations, from the early 1970s to late 2000s. *Note.* The authors used data from a Pew Research Center report (Taylor & Keeter, 2010)

As stated above, values and religiosity are connected (Roccas & Elster, 2013; Saroglou et al., 2004), and, therefore, a change in values that occurs between generations should also be reflected in changes in religiosity. For example, Gay et al. (2015) found that Generation X is a group that seeks spirituality, but not necessarily religiosity, whereas Generation Y is less religious than Baby Boomers but similar to Generation X in terms of engaging in religious practices within a specific tradition. It seems that generational affiliation may be connected to religiosity in the sense that the decline in religiosity is possibly related to generational patterns rather than age per se. This “cohort” theory (Firebaugh & Harley, 1991; Hout & Greeley, 1987) has received some support with analyses of big data sets conducted by the Pew Research Centre (Taylor & Keeter, 2010). Data gathered since the 1970s showed that differences in religiosity were significant between generations, but that religious attitudes within each cohort were relatively stable (Fig. 1). Additionally, to confirm these findings with another longitudinal demographic set, we explored data collected in the 32 subsequent waves of the General Social Survey (spanning the period from 1972 to 2018) in an independent analysis (see Supplemental Material for details). These results provide further support for the superiority of the cohort effect over the aging effect.

Given these findings, we expect more recent European generations to be less religious than earlier ones. We propose that this effect goes beyond the simple age differences between people from different generations and stems from generational shifts in human values.

### Overview of the Studies and Their Theoretical Contribution

This research tests the hypothesis that a generational shift in values explains the observed decline in religiosity beyond the effect of age. First, we expected that more recent generations would be less religious than earlier generations, and that this relationship would be independent of the developmental effect of age on religiosity. Second, with each subsequent generation, we expected a decline in social focus values, especially in conservation and, to a lesser extent, in self-transcendence, and a parallel increase in personal focus values, including openness to change and self-enhancement. Third, considering the results of the aforementioned meta-analysis (Saroglou et al., 2004), we expected that diminished conservation and increased openness to change values would predict a deterioration in the level of religiosity, and that this effect could be attributed to generational changes (above any effects of age found).

To verify our predictions, we first analyzed data from the seventh round of the European Social Survey (ESS7), conducted in 19 countries in 2014, and included participants representing three post-World War II generations: Baby Boomers, Generation X, and Generation Y. We tested whether four dimensions of basic human values—conservation, self-transcendence, openness to change, and self-enhancement—mediate the relationships between generational affiliation and religiosity. We found that each subsequent generation was less religious, and this effect could mainly be attributed to a shift from conservation values to openness to change values. However, because these data were collected at one point in time, it was impossible to disentangle the effect of age and the effect of generation in the analyses. The year of the participant’s birth determined age and their generational affiliation, and there was a full match between those two variables within one survey; that is, participants of the same age belonged to the same generation. Therefore, in the second step, we employed data from all rounds of the European Social Survey, from round 1 conducted in the period of 2002–2003 to round 9 conducted in the period of 2018–2019. This approach allowed us to partially separate age and generation. Year of the participant’s birth determined age and their generational affiliation, but because the time of the study changed, there was no longer a full match between those two variables across surveys; there were participants of the same age in subsequent surveys who belonged to different generations. We demonstrated that older people were less religious than younger people, and this effect was partially explained by the shift from openness to change values to conservation values. However, using two different operationalizations of generation, we found that generational decline in religiosity could also be attributed to generational increases in self-expansive, growth-based values constituting openness to change that was independent of the effect of age.

## Study 1

The aim of Study 1 was to provide preliminary data on the relationships between generations, human values, and religiosity. We analyzed data from ESS7, which had been conducted in 19 countries in 2014–2015. The survey included participants representing three post-World War II generations: Baby Boomers, Generation X, and Generation Y. We tested whether an increase in personal focus values such as openness to change and self-enhancement and a decrease in social focus values such as conservation and self-transcendence could account for the decrease in religiosity with each subsequent generation.

### Methods

#### Data

The analyses in this study were conducted using ESS round 7 (ESS7) data collected in the period of 2014–2015 (and published on December 1, 2018). There were a total of 40,185 participants in ESS7, living in 19 countries, including Austria, Belgium, Czechia, Denmark, Estonia, Finland, France, Germany, Hungary, Ireland, Israel, Lithuania, the Netherlands, Norway, Poland, Portugal, Slovenia, Spain, Sweden, Switzerland, and the UK. Of all the ESS7 participants, 53.98% were women, and 46.96% were men; 0.05% did not provide information on their gender. They ranged from 14 to 114 years in age (*M* = 49.28, *SD* = 18.74); 99 participants did not provide information on their year of birth. Samples were obtained through strict random probability methods to represent a population of all residents aged 15 years and older within private households, regardless of their nationality, citizenship, or language. Sample sizes ranged between 3045 (Germany) and 1224 (Slovenia). We considered data gathered from participants born between 1946 and 1997. Among a total of 32,350 potential participants for Study 2, 52.61% were women and 47.35% were men; 0.04% did not provide information on their gender. The age range of the potential participants was 17–69 years (*M* = 44.41, *SD* = 14.61). Not all potential participants provided answers to questions measuring our variables of interest; 1.97% did not answer one or more questions concerning religiosity, and 6.25% did not provide answers to one or more items measuring values. Therefore, we excluded 7.96% of ESS7 participants, leaving a final sample of 29,775 participants (52.89% women, 47.11% men; age: 17–69 years, *M* = 44.29, *SD* = 14.61).

### Measures

#### Religiosity

Individual religiosity was measured with three items: The first was on self-perceptions (“Regardless of whether you belong to a particular religion, how religious would you say you are?”). The response was based on an 11-point scale ranging from 0 (“not religious at all”) to 10 (“very religious”). The second was on frequency of participation in religious practices (“Apart from special occasions such as weddings and funerals, about how often do you attend religious services nowadays?”). The response was based on a 7-point scale ranging from 1 (“every day”) to 7 (“never”). The third was on frequency of personal praying (“Apart from when you are at religious services, how often, if at all, do you pray?”). The response was based on a 7-point scale from 1 (“every day”) to 7 (“never”). We recorded the answers so that a higher score reflected greater religious involvement for all three questions. The answers to the three questions were Z-scored and averaged to form a general indicator of religiosity (Cronbach’s α = 0.85).

#### Values

Values were measured using the Portrait Values Questionnaire (PVQ; Schwartz et al., 2001). The PVQ asks participants to compare themselves with 21 descriptions of people (“portraits”) and indicate how much they feel similar/dissimilar to each, using a 6-point scale from “very much like me” to “not like me at all” As we planned to analyze all dimensions in one statistical model, and since ipsative scores are linearly interdependent (Cheung, 2006), we decided not to transform raw data into ipsative scores but rather to calculate scores for each of the 10 value dimensions based on raw answers, and calculated the scores for four higher-order dimensions by averaging values scores (Schwartz, 2010). We calculated an average score for the basic values of conformity, security, and tradition for conservation; benevolence, tradition, and universalism for self-transcendence; stimulation, hedonism, and self-direction for openness to change; and power and achievement for self-enhancement.

#### Generation

We coded generation as a function of the year of birth of each participant, with Baby Boomers as participants born between 1946 and 1964 (*n* = 11,819; 53.26% women, 46.74% men; age 50–69, *M* = 59.26, *SD* = 5.50), Generation X as participants born between 1965 and 1976 (*n* = 7242; 53.60% women, 46.40% men; age 38–50, *M* = 44.00, *SD* = 3.51), and Generation Y as participants born between 1977 and 1997 (*n* = 10,714; 52,00% women, 48.00% men; age 17–38, *M* = 27.96, *SD* = 6.14). Detailed information on the sample is provided in Table 1.

**Table 1:** Study 1 descriptive statistics by generation

Variable	Baby Boomers (*n* = 11,819)		Generation X (*n* = 7242)		Generation Y (*n* = 10,714)	
	*M*	*SD*	*M*	*SD*	*M*	*SD*
Age of respondents	59.26	5.48	44.00	3.52	27.96	6.14
Religiosity	0.12	0.89	0.00	0.86	-0.13	0.85
Conservation	4.42	0.82	4.29	0.80	4.22	0.80
Self-transcendence	4.95	0.69	4.91	0.69	4.92	0.68
Openness to change	3.97	0.85	4.12	0.82	4.43	0.78
Self-enhancement	3.41	1.00	3.61	0.98	3.88	0.96

### Results and Discussion

Mediation analysis was conducted using Mplus 8.1 (Muthén et al., 2017). We used the robust full information maximum likelihood estimation method to estimate the assumed model of relationships with generational affiliation as an independent variable, four higher-order value dimensions as parallel operating mediators, and religiosity as the dependent variable. We also allowed for a correlation between the mediators. More specifically, we tested (1) the total effect of generation on the level of religiosity, (2) indirect effects of generation on the level of religiosity via each value dimension, controlling for the other dimensions, and (3) the total effect of generation on the level of religiosity controlling for four mediators. All continuous variables were Z-scored before the analysis to obtain the standardized coefficients. Because generation was a discrete ordinal variable with just three values, we used sequential coding (Hayes & Preacher, 2014). With sequential codes, the relative direct and indirect effects can be interpreted as the effect of membership in one group relative to the group one step sequentially lower in the ordered system. In our case, we used one dummy variable representing the comparison between Generation X and Baby Boomers, and a second dummy variable representing the comparison between Generation Y and Generation X. We examined the goodness of fit using multiple indices: the root mean square error of approximation (RMSEA) and 90% confidence interval (90% CI), standardized root mean square residual (SRMR), comparative fit index (CFI), and Tucker-Lewis index (TLI). We used multiple fit indices to assess different types of model fit (e.g., model parsimony and absolute fit), and for a more reliable and conservative evaluation when used together (Brown, 2006). The model was evaluated using the criteria proposed by Hu and Bentler (1999): the lower bound for a good fit was 0.95 for CFI and TLI, 0.06 for RMSEA, and 0.08 for SRMR. Indirect effects were tested by examining bootstrap confidence intervals for indirect effects using a bootstrap procedure with 10,000 samples.

The model fitted the data perfectly because it was saturated (RMSEA = 0, 90% CI [0, 0], SRMR < 0.001, TLI = 1, CFI = 1). All the hypothesized path coefficients were significant, except for the effect of Generation Y versus Generation X on self-transcendence. After excluding this insignificant path from the model, it fitted the data very well in light of all the examined indices (RMSEA = 0.005, 90% CI [0, 0.017], SRMR = 0.001, TLI = 0.99, CFI = 1). The results (standardized path coefficients) are presented in Table 2.

**Table 2:** Results of path analysis for Study 1

Structural paths	*β*	SE	*Z*	*p*
*Effects of generations on basic human values*				
Generation X versus Baby Boomers → Conservation	− 0.15	0.01	− 10.58	***
Generation X versus Baby Boomers → Self-transcendence	− 0.04	0.01	− 3.40	***
Generation X versus Baby Boomers → Openness to change	0.18	0.01	12.97	***
Generation X versus Baby Boomers → Self-enhancement	0.21	0.02	14.07	***
Generation Y versus Generation X → Conservation	− 0.01	0.01	− 7.05	***
Generation Y versus Generation X → Openness to change	0.35	0.01	25.60	***
Generation Y versus Generation X → Self-enhancement	0.26	0.02	17.59	***
*Effects of basic human values on religiosity*				
Conservation → Religiosity	0.24	0.01	36.78	***
Self-transcendence → Religiosity	0.04	0.01	6.34	***
Openness to change → Religiosity	− 0.14	0.01	− 20.98	***
Self-enhancement → Religiosity	0.02	0.01	3.44	***
Total effect of generation on religiosity				
Generation X versus Baby Boomers → Religiosity	− 0.14	0.02	− 9.12	***
Generation Y versus Generation X → Religiosity	− 0.14	0.02	− 9.39	***
*Direct effect of generation on religiosity*				
Generation X versus Baby Boomers → Religiosity	− 0.08	0.01	− 5.21	***
Generation Y versus Generation X → Religiosity	− 0.07	0.02	− 4.98	***
Relative indirect effects of generation on religiosity				
Generation X versus Baby Boomers via Conservation	− 0.04	0.004	− 10.17	***
Generation X versus Baby Boomers via Self-transcendence	− 0.002	0.001	− 3.00	***
Generation X versus Baby Boomers via Openness to change	− 0.03	0.002	− 11.03	***
Generation X versus Baby Boomers via Self-enhancement	0.01	0.001	3.34	***
Generation Y versus Generation X via Conservation	− 0.02	0.003	− 6.93	***
Generation Y versus Generation X via Openness to change	− 0.05	0.003	− 16.23	***
Generation Y versus Generation X via Self-enhancement	0.01	0.002	3.37	***

***p < .001

We observed significant effects of generations on all four value dimensions. For instance, Generation Xers, when compared with Baby Boomers, were less likely to endorse both social focus values (i.e., conservation and self-transcendence) and more likely to endorse both personal focus values (i.e., openness to change and self-enhancement). The effects for Generation Y in comparison with Generation X revealed a similar pattern, except that the path to self-transcendence was not significant. The total effects of generation on religiosity were significant, with Generation X being less religious than Baby Boomers, and Generation Y being less religious than Generation X. While controlling for mediators, the direct effects of generation remained significant, albeit weaker. The effect of conservation values on religiosity was significant and positive, similar to the effect of self-transcendence values, with the former being significantly stronger than the latter, *Z* = 17.97, *p* < 0.001. The effect of openness to change values on religiosity was significant and negative, whereas the respective effect of self-enhancement values was weak and positive. The predictors and mediators accounted for *R*^2^ = 9.1% of the variance in religiosity.

Further investigation of the 95% bootstrapped confidence intervals (95% bootCIs) for the relative indirect effects from generation to religiosity (see Table 2) confirmed significant indirect effects of Generation X (vs. Baby Boomers) on religiosity via conservation values, 95% bootCI [− 0.04, − 0.03] and via openness to change values, 95% bootCI [− 0.03, − 0.02], and indirect effects of Generation Y (vs. Generation X) on religiosity via conservation values, 95% bootCI [− 0.03, − 0.02] and via openness to change, 95% bootCI [− 0.05, − 0.03]. The indirect effects via self-enhancement and self-transcendence were much weaker (95% bootCI [0.002, 0.01] for the effect of Generation X [vs. Baby Boomers] on religiosity via self-enhancement, 95% bootCI [0.003, 0.01] for the effect of Generation Y [vs. Generation X] on religiosity via self-enhancement, and 95% bootCI [− 0.003, − 0.001] for the effect of Generation X [vs. Baby Boomers] on religiosity via self-transcendence), and their significance was most likely a result of the large sample size.

To summarize, using data from EES round 7, we demonstrated that Generation Y is less religious than Generation X, and Generation X is less religious than Baby Boomers. These differences in religiosity between generations are at least partially explained by generational differences in values, that is, lower endorsement of conservation values and higher endorsement of openness to change values in later generations compared with the previous ones. The results for self-enhancement and self-transcendence were inconclusive, with relatively weaker effects. This study provided preliminary support for our assumption that generational changes in values lead to a decrease in religiosity.

However, because we employed data that was collected at one point in time and coded generation as a function of year of birth, we could not separate the effect of generation from the effect of age; participants from later generations were not only born later, but they were also younger than participants from earlier generations. Furthermore, because of the relatively low representation of participants born before 1945 and after 1998 in the ESS7 sample, we had to limit our analysis to three generations only. Therefore, we decided to rerun our analysis using combined data from all rounds of the European Social Survey.

## Study 2

Study 2 used data from ESS rounds 1–9. The oldest data comes from ESS round 1, which was conducted in 2002–2003, while the newest data comes from round 9, conducted in 2018–2019. This approach allowed us to partially separate age and generation: the Baby Boomer born in 1962 would be around 40 years old during the first round of the survey, but 56 during the last round, while a person from Generation Y would be 24 during the first round and 40 years old during the last round. We were also able to include a reasonable number of Matures (people born between 1928 and 1945) and Generation Z (people born after 1998). We again tested whether an increase in personal focus values such as openness to change and self-enhancement and decrease in social focus values, such as conservation and self-transcendence, could account for the decrease in religiosity in subsequent generations. However, unlike in Study 1, we included both age and generation as separate predictors in the same statistical model to disentangle the specific effects of these two variables on basic human values and religiosity.

### Methods

#### Data

We analyzed data from all nine waves of the EES, including only countries that participated in all of these rounds. The initial dataset contained answers acquired from surveying 255,824 people living in 15 countries: Belgium, Finland, France, Germany, Hungary, Ireland, the Netherlands, Norway, Poland, Portugal, Slovenia, Spain, Sweden, Switzerland, and the UK. Among these EES participants, 52.57% were women, and 47.38% were men, and 0.06% did not provide information on their gender. Age at the time of the survey ranged from 14 to 123 years (*M* = 48.38 years, *SD* = 18.66), and 895 participants did not provide information on their year of birth. We considered data gathered from participants born between 1928 and 2004 (*N* = 246,603; 52.26% women, 47.72% men, 0.02% did not provide information on their gender; age at the time of the survey: 14–90, *M* = 47.19 years, *SD* = 17.72). Again, because not all participants provided answers to questions measuring our variables of interest—1.76% did not answer one or more question concerning religiosity, and 7.66% did not provide answer to one or more item measuring values—we excluded 9.04% of participants, leaving a final sample of 224,314 participants (52.28% women, 47.69% men, 0.02% did not provide information on their gender; age at the time of the survey: 14–90, *M* = 46.97 years, *SD* = 17.67).

### Measures

#### Religiosity

Individual religiosity was measured using the same three items used in Study 1. We recorded the answers so that a higher score reflected greater religious involvement for all three questions. The answers to the three questions were Z-scored and averaged to form a general indicator of religiosity (Cronbach’s α = 0.85).

#### Values

Again, values were measured using the PVQ (Schwartz et al., 2001). We calculated the scores for the four higher-order dimensions by averaging the raw values scores (Schwartz, 2010). We calculated an average score for the basic values of security and tradition for conservation; benevolence, tradition, and universalism for self-transcendence; stimulation, hedonism, and self-direction for openness to change, and power and achievement for self-enhancement.

#### Generation

In Study 2, generation was operationalized twofold. First, as in Study 1, we coded it as a function of the year of birth of each participant, with the Matures as those born between 1928 and 1945 (*n* = 41,640; 53.48% women, 46.49% men, 0.03% did not provide information on gender; age at the time of the survey 57–90 years, *M* = 71.37, *SD* = 6.65), Baby Boomers as those born between 1946 and 1964 (*n* = 75,214; 52.26% women, 47.71% men, 0.02% did not provide information on gender; age at the time of the survey 38–73 years, *M* = 54.99, *SD* = 7.56), Generation Xers as those born between 1965 and 1976 (*n* = 47,561; 52.63% women, 47.34% men, 0.02% did not provide information on gender; age at the time of the survey 26–55 years, *M* = 39.87, *SD* = 6.15), Generation Y as those born between 1977 and 1997 (*n* = 56,597; 51.31% women, 48.68% men, 0.01% did not provide information on gender; age at the time of the survey 14–43, *M* = 26.07, *SD* = 6.52), and Generation Z as those born after 1997 (*n* = 3302; 51.97% women, 49.03% men; age at the time of the survey 15–22, *M* = 17.28, *SD* = 1.63). Because there were more generations in Study 2 than in Study 1, and for the sake of brevity, in the analyses, we treated it as a scale variable, with larger values representing more recent generations. Descriptive statistics for the variables analyzed in this study are presented in Table 3, with age, religiosity, and higher-order values presented across the five generations.

**Table 3:** Study 2 descriptive statistics by generation

Variable	Matures (*n* = 41,640)		Baby Boomers (*n* = 75,214)		Generation X (*n* = 47,561)		Generation Y (*n* = 56,597)		Generation Z (*n* = 3302)	
	*M*	*SD*	*M*	*SD*	*M*	*SD*	*M*	*SD*	*M*	*SD*
Age of respondents	71.37	6.65	54.99	7.56	39.87	6.15	26.07	6.52	17.28	1.63
Religiosity	0.33	0.91	0.03	0.86	− 0.10	0.84	− 0.19	0.82	− 0.20	0.83
Conservation	4.57	0.78	4.31	0.82	4.20	0.81	4.12	0.80	4.07	0.78
Self-transcendence	4.90	0.68	4.92	0.66	4.91	0.65	4.91	0.65	4.95	0.66
Openness to change	3.77	0.85	3.99	0.81	4.17	0.79	4.46	0.76	4.62	0.71
Self-enhancement	3.25	0.96	3.36	0.96	3.56	0.95	3.81	0.93	3.87	0.90

Next, in a separate analysis (presented in the Supplemental Material), we also employed a different variable as a proxy for generation, which was coded based on the round of the ESS (1 to 9). The rationale behind this approach was that the sample for each round was representative of each society, and therefore, for earlier generations, there were larger numbers of participants in earlier rounds of the survey, and in later rounds the structure of the samples gradually changed, with more and more participants from recent generations (see Auxiliary Analysis for Study 2 in Supplemental Materials for details).

### Results and Discussion

As in Study 1, mediation analysis was conducted with Mplus 8.0 (Muthén et al., 2017) using the robust full information maximum likelihood estimation method. We tested a model of relationships with generational affiliation and age as independent variables, four dimensions of values as parallel operating mediators, and religiosity as the dependent variable. We also allowed for a correlation between the mediators. More specifically, we tested (1) the total effect of generation on religiosity, (2) indirect effects of generation on religiosity via each value dimension, controlling for the other dimensions, and (3) the total effect of generation on religiosity controlling for four mediators. All variables were Z-scored before the analysis to obtain the standardized coefficients.

The model fitted the data perfectly (RMSEA = 0, 90% CI [0, 0], SRMR < 0.001, TLI = 1, CFI = 1). All the hypothesized path coefficients were significant, except for the effect of generation on self-enhancement. After excluding this insignificant path from the model, it fitted the data very well in light of all the examined indices (RMSEA = 0.003, 90% CI [0, 0.007], SRMR < 0.001, TLI = 1, CFI = 1). The results (standardized path coefficients) are presented in Table 4.

**Table 4:** Results of path analysis for Study 2

Structural paths	*β*	SE	*Z*	*p*
*Effects of age on basic human values*				
Age → Conservation	0.23	0.005	45.34	***
Age → Self-transcendence	0.16	0.005	29.50	***
Age → Openness to change	− 0.21	0.005	− 44.42	***
Age → Self-enhancement	− 0.23	0.002	− 113.11	***
*Effects of generations on basic human values*				
Generation → Conservation	0.03	0.005	6.36	***
Generation → Self-transcendence	0.15	0.005	27.55	***
Generation → Openness to change	0.10	0.005	21.83	***
*Effects of basic human values on religiosity*				
Conservation → Religiosity	0.27	0.002	111.57	***
Self-transcendence → Religiosity	0.01	0.002	3.81	***
Openness to change → Religiosity	− 0.14	0.002	− 56.32	***
Self-enhancement → Religiosity	0.03	0.002	10.47	***
*Total effect on religiosity*				
Age → Religiosity	0.13	0.005	23.55	***
Generation → Religiosity	− 0.08	0.005	− 15.02	***
Direct effect on religiosity				
Age → Religiosity	0.04	0.005	7.53	***
Generation → Religiosity	− 0.08	0.005	− 14.77	***
*Relative indirect effects of age on religiosity*				
Age → Conservation → Religiosity	0.06	0.001	42.01	***
Age → Self-transcendence → Religiosity	0.001	0.0004	3.80	***
Age → Openness to change → Religiosity	0.03	0.001	34.88	***
Age → Self-enhancement → Religiosity	− 0.01	0.001	− 10.40	***
*Relative indirect effects of generation on religiosity*				
Generation → Conservation → Religiosity	0.01	0.001	6.35	***
Generation → Self-transcendence → Religiosity	0.001	0.0004	3.79	***
Generation → Openness to change → Religiosity	− 0.01	0.001	− 20.35	***

Generations coded based on year of birth

***p < .001

We observed significant effects of age on all four value dimensions, such as older participants who, when compared with younger participants, were more likely to endorse conservation and self-transcendence values, and less likely to endorse openness to change and self-enhancement values. Controlling for age, we found significant effects of generation on the three value dimensions. In line with our predictions, younger (later) generations were more likely to endorse openness to change values. However, contrary to our hypotheses, they were also more likely to endorse conservation and self-transcendence values. The total effect of age on religiosity was significant and positive. Controlling for age, the total effect of generation on religiosity was significant and negative. While controlling for mediators, the direct effect of age remained significant and positive, whereas the direct effect of generation remained significant and negative.

As in Study 1, the effect of conservation values on religiosity was significant and positive, similar to the effect of self-transcendence values, with the former being significantly stronger than the latter, *Z* = 63.65, *p* < 0.001. Again, as in Study 1, the effect of openness to change values on religiosity was significant and negative, and the effect of self-enhancement values on religiosity was weak and positive. The predictors and mediators accounted for *R*^2^ = 12% of the variance in religiosity.

Further investigation of the 95% bootCIs for the relative indirect effects of age on religiosity revealed significant indirect effects via conservation values, 95% bootCI [0.058, 0.064], and via openness to change values, 95% bootCI [0.028, 0.031]. The indirect effect via self-transcendence was positive but much weaker than the other effects, 95% bootCI [0.001, 0.002], and the effect of age on religiosity via self-enhancement was weak and negative, 95% bootCI [− 0.007, − 0.005]. The significance of the two latter indirect effects may be due to the large sample size and should be considered with caution. The overall indirect effect of age on religiosity via human values was significant and positive, *β* = 0.09, SE = 0.002, *Z* = 49.29, *p* < 0.001, 95% CI [0.08, 0.09].

Finally, we investigated the 95% bootCIs for the relative indirect effects from generation to religiosity. In line with our expectations, the indirect effect of openness to change was significant and negative. The indirect effects via conservation and self-transcendence were positive but weaker than the other indirect effects. The overall indirect effect of generation on religiosity via human values was significant and negative, *β* = − 0.004, SE = 0.002, *Z* = − 2.81, *p* < 0.001, 95% CI [− 0.01, − 0.001].

To summarize, using the data from multiple rounds of the ESS, we were able to independently analyze the effect of age and generation on human values and religiosity in the same model. We demonstrated that, after controlling for generation, age was positively related to religiosity. This means that within the same generation, older people were more religious than younger ones. However, this effect was accompanied by a negative effect of generation after controlling for age, indicating that two people of the same age but coming from different generations (thus, surveyed in different waves of ESS [e.g., a 19-year-old in round 1 vs a 19-year-old in round 2) would also differ in terms of religiosity, with members of more recent generations being less religious than members of older ones. Furthermore, the effects of age and generation were mediated by basic human values. Older people were more prone to endorse conservation values and less prone to endorse openness to change values, which led to higher religiosity. However, we also observed a unique indirect effect of generation via openness to change on religiosity, present for two different operationalizations of generation (see Supplemental Materials for additional analyses). People from more recent generations were more open to change than those from less recent ones, which in turn led to lower levels of religiosity, and this effect was present over and above the effect of age on religiosity both directly and via basic human values. Interestingly, when controlling for age, we observed a positive—and not negative as expected—effect of membership in a younger generation on self-transcendence. However, this effect translated into increased religiosity, with an extremely small effect size. Finally, we also systematically did not observe the effect of generation on religiosity via decreased conservation values. In fact, we found some increase in conservation in more recent generations when compared with the older ones, but this increase translated into only slight proliferation of religiosity. Hence, the increase in conservation values leading to higher religiosity should be attributed to age differences rather than to generational affiliation, while the alterations in openness to change affecting religiosity could be attributed both to age (with older people being less open to change and therefore more religious than younger people) and generational differences (with more recent generations being more open to change and therefore less religious than older generations).

## General Discussion

We started this study with the rather paradoxical observation that even though societies are aging and age is positively related to religiosity, there was a decrease rather than an increase in religiosity. We aimed to identify a mechanism explaining this decline in religiosity that would work above and beyond the positive relationship between age and religiosity. In search of this mechanism, we considered the construct of basic human values, as described by Schwatz (1992), and the evolution of values across consecutive generations.

To make a clear distinction between these two factors, (age and generational affiliation) we analyzed data from a Pew Research Report (Taylor & Keeter, 2010), and conducted our own analysis on general social survey data (preliminary study in Supplemental Material). The results of both these analyses suggested that generational affiliation has a distinct effect on religiosity that is independent of age-related changes. The intergenerational change in religiosity appears to be strong, whereas within a single generation, religiosity variation over the life span is much smaller.

We further explored the nature of this age-generation-religiosity relationship, with the mediating role of changes in human values. In two studies based on data from the ESS (round 7 for Study 1 and rounds 1–9 for Study 2), we tested whether a decline in religiosity could indeed be explained by a shift in values that occurred across generations after World War II, mainly from conservation toward openness to change, and from self-transcendence toward self-enhancement. In Study 1, we demonstrated that Generation Y is less religious than Generation X, while Generation X is less religious than Baby Boomers. These differences in religiosity between generations were at least partially explained by a generational shift in values, from conservation to openness to change and, to a smaller extent, from self-transcendence to self-enhancement. Nonetheless, data collected at one point in time did not allow the separation of the effect of generation from the effect of age. Hence, in Study 2, we retested our hypotheses using combined data from all rounds of ESS. First, we found that age and generation were independently related to religiosity. Older people were indeed more religious than younger people, but also, when controlling for age, more recent generations were less religious than the previous ones. We also found evidence supporting the claim that as people age, they tend to shift their focus from openness to change to conservation values, and from self-enhancement to self-transcendence, and this change in personal values transfers to proliferation in religiosity. Most importantly, we also found an indirect negative effect of generation on religiosity via openness to change that goes beyond and above the effect of age. People from more recent generations were more open to change than those from older ones, which in turn led to lower levels of religiosity.

In addition to the results that were consistent with our research hypotheses, some of our findings were unexpected and surprising. First, in both studies, we found a positive, though very weak, relationship between self-enhancement and religiosity that replicated previous findings. Self-enhancement values, such as power, achievement, and hedonism, have always been described as negative correlates of religiosity, and this was confirmed by a meta-analysis conducted by Saroglou et al. (2004). However, since age also correlates negatively with self-enhancement (Robinson, 2013), it is possible that these negative correlations found earlier are only statistical artifacts stemming from the fact that age was not controlled for in the analysis, and would disappear when controlling for age, or, as we demonstrated, even become weakly positive.

When controlling for age in Study 2, we also found a positive, and not negative as expected, effect of more recent generations on self-transcendence that only weakly translates to an increase in religiosity. It seems that people from younger generations may endorse self-transcendence values more, but they may act upon them outside of an organized religion or mainstream church. Indeed, it has been demonstrated that members of Generation X and Y are more likely to seek spirituality rather than religiosity when compared with Baby Boomers (Gay et al., 2015). It is possible that they find other ways and philosophies that sustain their self-transcendent beliefs that are related to spirituality or secular institutions, such as non-governmental organizations aimed at helping others, caring for the environment, and promoting tolerance (Mason et al., 2007).

Furthermore, we did not observe the effect of generation on religiosity via decreased conservation values, and found some increase in conservation in more recent generations when compared with the older ones, but this increase led to only weak proliferation of religiosity. It also seems that people from younger generations find ways of acting upon their conservation values outside of mainstream religious traditions. With regard to the effects related to self-transcendence, they may seek security within secular groups or organizations and conform to their norms and rules rather than those dictated by religious authorities. If this is the case, then religiosity decline may have a snowball dynamic. With its decreasing role in social life, the values that were related to higher religiosity among earlier generations may no longer support it. As being religious becomes less of a norm, ways of expressing conformity and seeking security become more secular, and this again translates into religion becoming even less of a norm. One such norm that gains popularity in young generations that can serve self-protection needs is national populism, which is also a powerful form of collective identity, not necessarily related to strong religious identification (Rieffer, 2003).

Each successive generation after World War II was exposed to different standards of socialization and values, which, in turn, could influence their motives for engaging in religious practices. Religiosity, as we describe, is univariate, measured as a self-reported identity (self-perception question about being religious), and via behavioral indicators (attending worship services and frequency of prayer). However, numerous studies have indicated that religiosity should always be studied according to its functional types. One of the first such concepts was Allport’s theory (Allport, 1966), which distinguished intrinsic from extrinsic religiosity. This theory was later expanded by Batson (1976), who added a third type: quest religiosity. By their very definitions, these three types of religiosity seem to be strongly embedded in completely different motives and human values (Gennerich & Huber, 2006). For intrinsically focused believers, religion is the ultimate end in itself, and involves upholding values such as humility and compassion. Extrinsically focused believers have a utilitarian view of religion. For these believers, religion is a way of being a part of a social group, and fulfills security, identity, belonging, and social support needs (Nezlek, 2020). Both intrinsic and extrinsic religiosity are more conservative and authoritarian forms of religiosity and are immanently related to power and security values. In contrast, those for whom the “quest” aspect of religiosity is dominant, do not hesitate to doubt, ask questions, and search for alternative explanations. This type of religiosity is strongly related to environmentalism, prosocial orientation, low prejudice, and universalism (Gennerich & Huber, 2006). Reducing the concept of religiosity to a one-dimensional approach seems to be a considerable limitation of the presented research results, as each type of religiosity might be influenced by values in a completely different way (and with a different strength). The ESS data did not allow for such analyses, and further research that considers different types of religiosity is warranted.

Our results provide new knowledge and insight on the psychological predictors of religious attitudes. It is highly probable that generation-related changes in religiosity are explained by changes in the structure of human values, namely an increase in hedonism, stimulation, and self-direction, constituting openness to change. However, the generational shift in values explains only a small part of the decline in religiosity, and numerous other social, cultural, and contextual factors may play a role in this process (Greeley, 2017). Rachmatullah et al. (2019) revealed in their multinational study that religiosity is strongly related to education and socioeconomic status (continually increasing in modern Europe). As education improves and socioeconomic status increases, people acquire cognitive and material means to cope with life’s challenges with a greater sense of agency, so they may no longer feel the need to turn to religion for support.

### Limitations

One major limitation of our study is its cross-sectional design, which did not allow us to draw causal inferences about the relationships between our variables of interest. The mediation model was built on the assumption that value structure, which is closely related to basic personality traits and people’s conscious goals (Fischer, 2018), affects levels of religiosity; however, this relationship is bidirectional (Chan et al., 2018; Roccas & Elster, 2013). On the one hand, people may feel motivated to belong to groups with shared social goals (Gandal et al., 2005), and an important aim of religions is to shape its members’ values according to its ideology (McCullough & Willoughby, 2009). Unfortunately, the ESS did not include the same participants in each round, so it does not provide longitudinal data at the individual level that would at least partially allow us to answer the question of causality. Future research should collect longitudinal data to better understand the causality of the investigated effects.

Another important limitation of our study is that the list of countries participating in the ESS is rather limited, focusing on countries strongly embedded in the European tradition. The advantage of this dataset may be the strong cross-country variation in religiosity (from very religious Poland to strongly secularized Czechia), while the undoubted disadvantage is its geographical and cultural homogeneity. Therefore, our findings can only be generalized to other countries with similar cultures or European origin (e.g., North America), but one should be very cautious when using these data in highly collectivistic countries. Furthermore, we did not investigate the country-specific relationships between age, generation, values, and religiosity. However, the strength of the relationship between residents’ values and individual religiosity may differ depending on the general level of religiosity in a specific country. On the one hand, in highly religious societies, socially oriented people may be naturally motivated to identify as religious, because they then reach a person-environment congruence in values known to be associated with better well-being (Sortheix & Schwartz, 2017). In addition, this effect can be explained in light of the aforementioned terror management theory (Greenberg et al., 1986), which describes ways of coping with the perspective of inevitable death. In highly religious societies, where believing in God and the afterlife is the most common way of reacting to the salience of mortality, conservation values (tradition, conformity, and security) are core elements of social identity. Therefore, people high in those values try to fulfill them through a socially activated worldview: religion. On the other hand, in highly secularized countries, relationships between values and religious affiliation may be much weaker, presumably because of other (non-religious) foundations underlying people’s beliefs and goals. Confirming self-esteem (related to achievement and power) is the most common method of coping with death anxiety among non-religious people (Kashima et al., 2004), and the openness to change values such as hedonism, stimulation, self-direction are more likely to dominate in highly individualistic cultures (Cukur et al., 2004). In secularized countries, conservation values may poorly reflect individual religiosity, mainly because of different patterns of social identity bonds. In their meta-analysis on the relationship between human values and religiosity, Saroglou et al. (2004) demonstrated that the more developed a country, the less positive the correlation of religiosity with conservation, and the less negative the correlation of religiosity with self-direction and achievement. Because country religiosity declines as material security and quality of life improve (Barber, 2013), this may also translate into diverse relations between values and religiosity in different countries. Hence, future research should investigate the relationship between age, generation, values, and religiosity, not only at the individual level, but also taking country specificity into account.

## Conclusions

The undeniable strengths of the analyses presented here are the enormous sample sizes, collected within the ESS with great care, and in compliance with the principles of representativeness for the population. Because of this data richness, along with the availability of multiple waves in this survey collected over almost 20 years, the picture of changes in the European value structure is accurate and realistic.

Sociologists often suggests that the causes of negative changes in religion, politics, or family functioning is a *crisis of values* (Ebaugh, 2007). In this study, we avoided explicit assessment of the observed changes, showing instead their connection with the evolution of religiosity over the past few generations. We demonstrate that starting with the generation born before 1928, there is a clear trend of decreasing emphasis on conservation values, and increasing emphasis on openness to change, responsible for the reduction in religiosity in Europeans. This mechanism appears to be stronger than the age-related changes in religiosity, which, in the context of a clearly aging population, should lead not to a decline, but to an increase in the importance of religion in Europe.

The results of our study are focused on the past, but it appears that they might be the key to discussions about the future of religion and spirituality in Europe. Knowledge on the structure of values in the current group of the youngest Europeans allows the prediction of dominant European values in several decades, when today's youth will become the most influential group. Such analyses are also possible due to the observation and analysis of factors that characterize generations (e.g., primary media sources or cultural preferences). In our opinion, knowledge on the interdependencies between generational affiliation, changes in value structure, and religiosity can serve as an important basis for further consideration of European faith and spirituality.

### Supplementary Information

Below is the link to the electronic supplementary material.

Supplementary file1 (DOCX 187 kb)
