# Supplementary material for: Religiosity Decline in Europe: Age, Generation, and the Mediating Role of Shifting Human Values
Source: J Relig Health. 2022 Sep 23;63(2):1091–116. doi: 10.1007/s10943-022-01670-x (PMC10965724; doi:10.1007/s10943-022-01670-x)
Supplement: Supplementary file 1 — Supplementary file1 (DOCX 187 kb) [file 10943_2022_1670_MOESM1_ESM.docx]

**Preliminary study**

In a preliminary study, we analyzed data from the General Social Survey (GSS; National Opinion Research Center, 2019), a survey regularly collected since 1972 by the National Opinion Research Center at the University of Chicago on representative samples of residents of the United States. We aimed to collect initial evidence on whether later generations would be less religious than earlier generations, and whether this result was independent of the developmental effect of age on religiosity.

# Methods

***Data***

The GSS uses full-probability sampling to select respondents from an adult, English-speaking, noninstitutionalized population of the United States. In this study, we employed data from all 32 rounds of the GSS conducted between 1972 and 2018. Therefore, the initial dataset contained answers acquired from surveying 64,814 people (55.85% women, 44.15% men; age at the time of survey: 18+, *M* = 46.10 years, *SD* = 17.53; 228 participants did not provide information on their age). We considered data gathered from participants born between 1928 and 1997 (*N* = 52,847; 55.01% women, 49.99% men; age at the time of survey: 18+, *M* = 41.18 years, *SD* = 14.63). We excluded 0.86% of participants who did not answer the question measuring the frequency of attending religious services, leaving a final sample of 52,390 individuals (55.02% women, 49.98% men; age at the time of survey: 18+, *M* = 41.16 years, *SD* = 14.63).

***Measures***

Religiosity. Individual religiosity was measured with the question concerning the frequency of participation in religious practices (“How often do you attend religious services?” with responses based on a scale from 0 = “never” to 8 “more than once a week”).

**Generation**. We operationalized generation as a function of the year of birth of each participant, with Matures as those born between 1928 and 1945 (*n* = 13,775; 55.85% women, 44.15% men; age at the time of survey 27-89 years, *M* = 53.86, *SD* = 13.86), Baby Boomers as those born between 1946 and 1964 (*n* = 23,685; 54.55% women, 54.55% men; age at the time of survey 18-72 years, *M* = 40.41, *SD* = 12.54), Generation X as those born between 1965 and 1976 (*n* = 8,896; 54.84% women, 45.16% men; age at the time of survey 18-53 years, *M* = 33.15, *SD* = 8.41); and Generation Y as those born between 1977 and 1997 (*n* = 6,034; 55.22% women, 44.78% men; age at the time of survey 18-41, *M* = 26.95, *SD* = 5.58).

**Results**

In the preliminary analysis, there was a significant and positive correlation between age and religiosity, *r* = .09, *p* < .001, indicating that older participants attend religious services more often than younger ones. There was also a positive correlation between the year of data collection and participant age, *rho* = .35, *p* < .001, indicating that Americans are aging; the mean age in the representative sample increased from 1970. Finally, there was a negative correlation between the year of data collection and religiosity, *rho* = -.06, *p* < .001, indicating that although the U.S. population is aging, and although older people are more religious, the level of religiosity in the representative sample decreases rather than increases.

To disentangle this paradox, we conducted an analysis of variance with generation as a nominal factor (Matures vs. Baby Boomers vs. Generation X vs. Generation Y), age as a continuous predictor, and the frequency of participation in religious practices as a dependent variable. We found a significant main effect of generation, *F*(3, 52,382) = 10.25, *p* < .001, η^2^ = .001; for example, the level of religiosity was almost linearly decreasing from generation to generation, *M* = 4.10, *SD* = 2.76 for Matures, *M* = 3.60, *SD* = 2.67 for Baby Boomers, *M* = 3.36, *SD* = 2.63 for Generation X and *M* = 2.9, *SD* = 2.60, each pair of means significantly different at *p* < .05 (see Figure S1). The main effect of age was also significant, *F*(1, 52,382) = 6.85, *p* < .001, η^2^ < .001, with older participants being slightly more religious than younger ones. The interaction between these two variables was not significant, *F*(3, 52,382) = 1.11, *p* = .341, η^2^ < .001, indicating that the differences in religiosity between generations were independent of participants’ age at the time of the study.


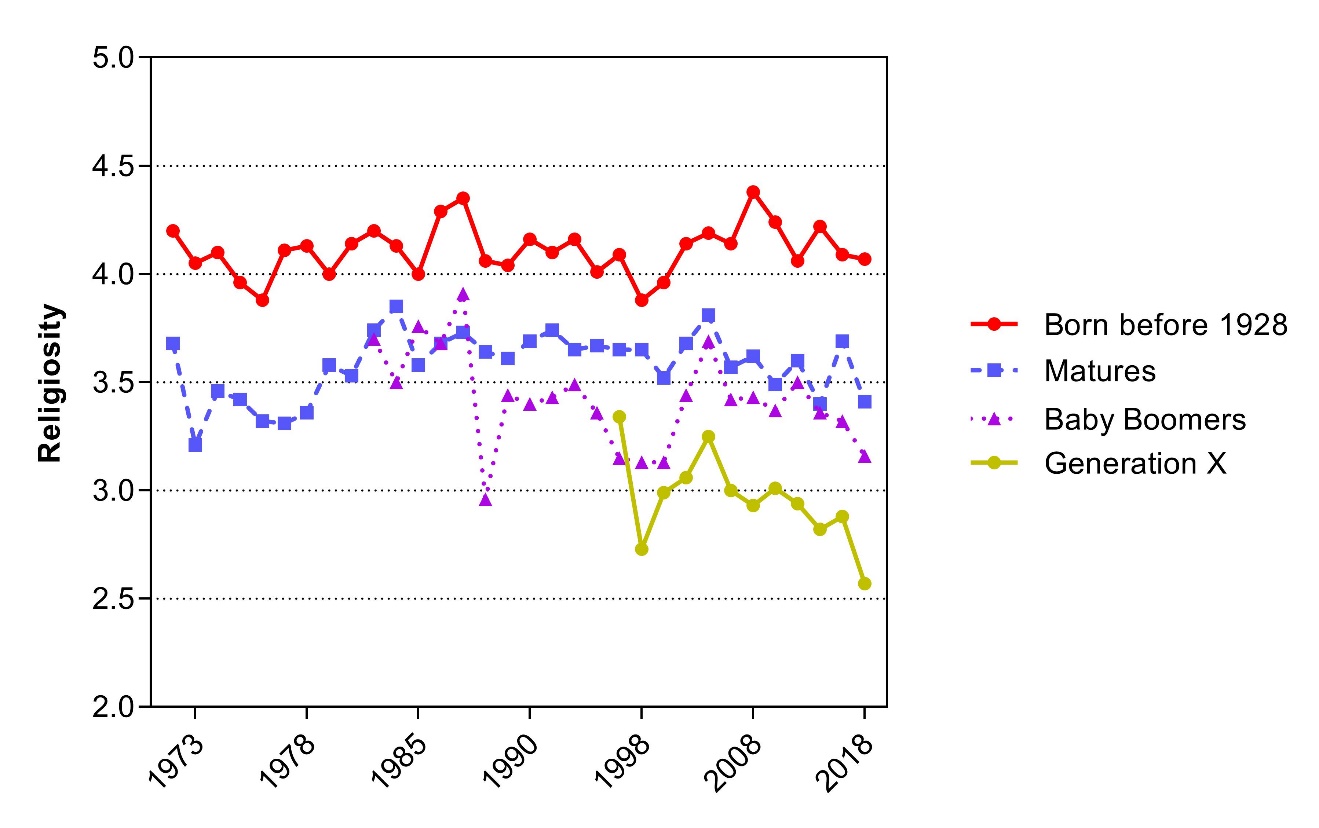


Figure S1*.* Changes over time in religious affiliation in successive U.S. generations, from 1972 to 2018. Prepared by the Authors using data from the General Social Survey.

In conclusion, in our analysis of cross-sectional data from more than 30 rounds of the GSS, collected over the course of almost 50 years, we found a higher religiosity in older people compared with younger people may be more a result of differences in cohorts than the developmental effect of age.

**Study 2**

**Auxiliary analysis**

***Generation coding based on the European Social Survey round***

We assumed that the round of the European Social Survey (ESS; from round 1 in 2002 to round 9 in 2018) could be treated as another proxy for generation. The rationale behind this approach was that the sample for each round represented the population at that time. Therefore, there were more members of earlier generations in earlier rounds of the survey. In later rounds, the structure of the samples gradually changed, with later generations being more and more widely represented (see Table S1). In other words, this operationalization allowed for the disentanglement of the factors of age and generational affiliation, making it possible to estimate the independent association of each of these variables with changes in religiosity.

**Table S1**

*Generational representation in each of the ESS rounds*

| ESS round | Statistics | Matures  (0) | Baby Boomers  (1) | Generation X  (2) | Generation Y  (3) | Generation Z  (4) | TOTAL |
| --- | --- | --- | --- | --- | --- | --- | --- |
| 1 | Count | 6679 | 9964 | 6026 | 4437 | 0 | 27106 |
|  | % within row | 24.64 % | 36.76 % | 22.23 % | 16.37 % | 0 % | 100 % |
| 2 | Count | 6463 | 9383 | 5959 | 5165 | 0 | 26970 |
|  | % within row | 23.96 % | 34.79 % | 22.09 % | 19.15 % | 0 % | 100 % |
| 3 | Count | 6258 | 9346 | 5952 | 5698 | 0 | 27254 |
|  | % within row | 22.96 % | 34.29 % | 21.84 % | 20.91 % | 0 % | 100 % |
| 4 | Count | 5991 | 9225 | 6040 | 6766 | 0 | 28022 |
|  | % within row | 21.38 % | 32.92 % | 21.55 % | 24.15 % | 0 % | 100 % |
| 5 | Count | 5374 | 8991 | 5747 | 7573 | 0 | 27685 |
|  | % within row | 19.41 % | 32.48 % | 20.76 % | 27.35 % | 0 % | 100 % |
| 6 | Count | 5243 | 9576 | 6041 | 8480 | 8 | 29348 |
|  | % within row | 17.86 % | 32.63 % | 20.58 % | 28.89 % | 0.03 % | 100 % |
| 7 | Count | 4524 | 9100 | 5417 | 7871 | 579 | 27491 |
|  | % within row | 16.46 % | 33.10 % | 19.70 % | 28.63 % | 2.11 % | 100 % |
| 8 | Count | 3714 | 8883 | 5509 | 7897 | 1184 | 27187 |
|  | % within row | 13.66 % | 32.67 % | 20.26 % | 29.05 % | 4.36 % | 100 % |
| 9 | Count | 3208 | 8099 | 4998 | 7497 | 1738 | 25540 |
|  | % within row | 12.56 % | 31.71 % | 19.57 % | 29.35 % | 6.81 % | 100 % |
| TOTAL | Count | 47454 | 82567 | 51689 | 61384 | 3509 | 246603 |
|  | % within row | 19.24 % | 33.48 % | 20.96 % | 24.89 % | 1.42 % | 100 % |

As in the main study, we conducted a mediation analysis with MPlus 8.0 (Muthén et al., 2017) using the robust full information maximum likelihood estimation method. We tested a model of relationships with generation affiliation and age as independent variables, four dimensions of values as parallel operating mediators, and religiosity as the dependent variable. We also allowed for a correlation between the mediators. More specifically, we tested (1) the total effect of generation on level of religiosity, (2) the indirect effects of generation on the level of religiosity via each value dimension, controlling for the other dimensions, and (3) the total effect of generation on the level of religiosity controlling for four mediators. All variables were Z-scored before the analysis in order to obtain the standardized coefficients.

The model fitted the data perfectly again (*RMSEA* = 0, 90% *CI* [0, 0], *SRMR* < .001, *TLI* = 1, *CFI* = 1). All the hypothesized path coefficients were significant, except for the effect of generation on conservation. After excluding this insignificant path from the model, the model fitted the data very well in light of all the examined indices (*RMSEA* < .001, 90% *CI* [0, < .001], *SRMR* < .001, *TLI* = 1, *CFI* = 1). The standardized path coefficients are presented in Table S2 in the Supplemental Material.

Again, we observed significant effects of age on all four value dimensions. Compared with younger participants, older participants were more likely to endorse conservation and self-transcendence values and were less likely to endorse openness to change and self-enhancement values. Controlling for age, we found significant effects of rounds as a proxy for generation on three value dimensions. In line with our predictions, compared with earlier rounds, participants from later rounds were more likely to endorse openness to change values. However, contrary to our hypotheses, they were also less likely to endorse self-enhancement values and self-transcendence values. The total effect of age on religiosity was significant and positive. Controlling for age, the total effect of rounds on religiosity was significant and negative, confirming the decline in religiosity over the course of the last century. While controlling for mediators and rounds, the direct effect of age remained significant and positive. While controlling for mediators and age, the direct effect of rounds remained significant and negative.

As in Study 1 and in the analysis with generation coded on the basis of age for Study 2 (presented in the main text), the effect of conservation values on religiosity was significant and positive, similar to the effect of self-transcendence values, with the former being significantly stronger than the latter, *Z* = 63.65, *p* < .001. Again, the effect of openness to change values on religiosity was significant and negative, while the effect of self-enhancement values on religiosity was weak and positive. The predictors and mediators accounted for *R*^2^ = 12.30% of the variance in religiosity.

**Table S2**

*Results of additional path analysis for Study 2. Generation coded as the round of ESS*

| Structural paths | β | *se* | *Z* | *p* |
| --- | --- | --- | --- | --- |
| *Effects of age on basic human values* |  |  |  |  |
| Age 🡪 Conservation | .20 | .002 | 97.13 | *** |
| Age 🡪 Self-transcendence | .01 | .002 | 6.08 | *** |
| Age 🡪 Openness to change | -.31 | .002 | -154.72 | *** |
| Age 🡪 Self-enhancement | -.23 | .002 | -112.24 | *** |
| *Effects of generation on basic human values* |  |  |  |  |
| Generation 🡪 Self-transcendence | .09 | .002 | 45.40 | *** |
| Generation 🡪 Openness to change | .06 | .002 | 30.62 | *** |
| Generation 🡪 Self-enhancement | -.01 | .002 | -2.47 | * |
| *Effects of basic human values on religiosity* |  |  |  |  |
| Conservation 🡪 Religiosity | .26 | .002 | 110.91 | *** |
| Self-transcendence 🡪 Religiosity | .01 | .002 | 5.331 | *** |
| Openness to change 🡪 Religiosity | -.14 | .002 | -55.60 | *** |
| Self-enhancement 🡪 Religiosity | .02 | .002 | 9.98 | *** |
| *Total effect on religiosity* |  |  |  |  |
| Age 🡪 Religiosity | .21 | .002 | 99.15 | *** |
| Generation 🡪 Religiosity | -.07 | .002 | -32.75 | *** |
| *Direct effect on religiosity* |  |  |  |  |
| Age 🡪 Religiosity | .12 | .002 | 52.87 | *** |
| Generation 🡪 Religiosity | -.06 | .002 | -29.10 | *** |
| *Relative indirect effects of age on religiosity* |  |  |  |  |
| Age 🡪 Conservation 🡪 Religiosity | .05 | <.001 | 73.07 | *** |
| Age 🡪 Self-transcendence 🡪 Religiosity | <.001 | <.001 | 4.01 | *** |
| Age 🡪 Openness to change 🡪 Religiosity | .04 | .001 | 52.32 | *** |
| Age 🡪 Self-enhancement 🡪 Religiosity | -.01 | .001 | -9.94 | *** |
| *Relative indirect effects of generation on religiosity* |  |  |  |  |
| Generation 🡪 Self-transcendence 🡪 Religiosity | <.001 | <.001 | 5.30 | *** |
| Generation 🡪 Openness to change 🡪 Religiosity | -.01 | .000 | -26.82 | *** |
| Generation 🡪 Self-enhancement 🡪 Religiosity | -.0001 | <.001 | -2.40 | * |

Further investigation of the 95% bootstrapped confidence intervals (BootCIs) for the relative indirect effects of age on religiosity revealed significant indirect effects via conservation values, 95% BootCI [.051, .054], and via openness to change values, 95% BootCI [.041, .045]. The indirect effect via self-transcendence was positive but much weaker than the other effects, 95% BootCI [ .0001, .0002], and the effect of age on religiosity via self-enhancement was weak and again negative, 95% BootCI [-.007, -.004]. Again, the significance of the two latter indirect effects may be due to the large sample size and therefore, this finding should be viewed with caution. The overall indirect effect of age on religiosity via human values was significant and positive, β = .09, *se* = .001, *Z* = 84.04, *p* < .001, 95% CI [.088, .093].

Finally, we investigated the 95% BootCIs for the relative indirect effects from rounds as a proxy for generation to religiosity. In line with our expectations, the indirect effect via openness to change values was significant and negative, β 95% BootCI [-.009, -.008]. The indirect effects via self-transcendence and self-enhancement were much weaker than the other effects, 95% BootCI [ .0007, .001] for self-transcendence, and 95% BootCI [-.0002, -.00002] for self-enhancement. The overall indirect effect of round on religiosity via human values was significant and negative, β = -.01, se < .001, *Z* = -22.82, *p* < .001, 95% CI [-.008, -.007].
